# 免疫检查点抑制剂相关心肌炎的研究进展

**DOI:** 10.3779/j.issn.1009-3419.2021.102.27

**Published:** 2021-09-20

**Authors:** 运炜 刘, 燕欣 陈, 治民 曾, 安文 刘

**Affiliations:** 330006 南昌，南昌大学第二附属医院肿瘤科，江西省肿瘤临床转化重点实验室 Department of Oncology, The Second Affiliated Hospital of Nanchang University, Jiangxi Key Laboratory of Clinical Translational Cancer Research, Nanchang 330006, China

**Keywords:** 免疫检查点抑制剂, 心肌炎, 心脏毒性, Immune checkpoint inhibitor, Myocarditis, Cardiotoxicity

## Abstract

免疫检查点抑制剂（immune checkpoint inhibitors, ICIs）是一种负性调节因子抗体，激活T细胞发挥免疫治疗抗肿瘤作用的同时，也可引起免疫相关的不良应答，从而诱导出一系列免疫相关不良反应（immune related adverse events, irAEs）。在这些irAEs中，ICIs相关心肌炎的发病率虽然很低，但致死率却明显高于其他不良反应，接近50%，临床医生应用ICIs时应提高警惕，但目前ICIs相关心肌炎的发病机制仍不明确。本文结合近期ICIs的研究成果对ICIs相关性心肌炎的发生机制及临床表现等方面作一综述，以提高临床医生对该不良反应的认识。

## Abstract (en)

Immune checkpoint inhibitors (ICIs) is a negative regulatory factor antibody, which activates T cells to play an anti-tumor effect in immunotherapy, and can also cause immune-related adverse responses, thereby inducing a series of immune related adverse events (irAEs). Among these irAEs, although the incidence of ICIs-related myocarditis is very low, the fatality rate is significantly higher than other adverse reactions, close to 50%. Clinicians should be vigilant when applying ICIs, but the pathogenesis of ICIs-related myocarditis is still unclear. This article combines the recent research results of ICIs to summarize the mechanism and clinical manifestations of ICIs-related myocarditis, so as to improve clinicians' understanding of the adverse reactions.

近年来，免疫检查点抑制剂（immune checkpoint inhibitors, ICIs）在黑色素瘤、肾细胞癌、非小细胞肺癌等多种肿瘤中取得了令人瞩目的疗效，改变了传统肿瘤治疗策略，为患者带来了更多的生存获益。目前，免疫治疗已成为恶性肿瘤治疗中最具潜力的策略之一。ICIs治疗引发的脱靶效应和抗肿瘤作用之间关系密切，ICIs通过解除免疫抑制、活化T细胞功能来逆转免疫耐受，增强T细胞抗肿瘤反应；同时，过度活化的T细胞通过攻击正常组织、产生自身抗体、增加细胞因子等诱发自身免疫不良事件，打破了正常免疫系统的平衡，产生一系列免疫治疗相关不良反应（immune related adverse events, irAEs）。irAEs可累及全身各个系统或器官，主要涉及胃肠道、内分泌和皮肤，而中枢神经系统、心血管系统、呼吸系统、肌肉骨骼系统和血液系统则累及较少。其中ICIs相关心肌炎的发病率虽低，但致死性较高，且其发生机制尚不清楚。因此，irAEs是免疫治疗中需攻克的关键难题，深入研究irAEs的分子机制意义重大。现着重介绍ICIs相关心肌炎的流行病学、病理生理学机制、临床表现和诊断。

## 1 ICIs相关心肌炎流行病学

虽然ICIs相关心肌炎发生率为0.09%-1.14%，但由于严重心肌炎病死率高，已成为威胁接受ICIs治疗患者生命最主要的毒性之一^[1-3]^。2018年通过检索世卫组织全球个例安全数据库VigiBase，开展了一项评估ICIs与心脏毒性之间关系的观察性、回顾性、药物警戒性研究，统计了31, 321例发生了免疫不良事件的病例，发现10.28%（3, 219例）具有心脏毒性，其中心肌炎发生率为0.39%（122例），单用程序性死亡受体1（programmed cell death 1, PD-1）抑制剂或程序性死亡受体配体1（programmed cell death ligand 1, PD-L1）抑制剂、细胞毒T淋巴细胞抗原4（cytotoxic T lymphocyte antigen 4, CTLA-4）抑制剂时心肌炎的发生率分别为0.41%、0.07%，而两种ICIs联合治疗时心肌炎的发生率为1.3%^[4]^。

尽管ICIs相关心肌炎很少见，但其致死性高达50%，其中PD-1抑制剂联合CTLA-4抑制剂引起的心肌炎患者致死率甚至高达67%，而心脏毒性中心包炎、血管炎的致死率分别为21%、6%，显著低于心肌炎的致死率^[2, 4, 5]^。

发生心肌炎的时间因病史、检查点抑制剂类型、应用时间以及双药或单药而异。据既往文献报道，ICIs相关心肌炎的发生贯穿于免疫治疗全程，首次应用ICIs后即可发病，大约80%的ICIs相关心肌炎发生于首次治疗后3个月内，但也有迟发性起病的报道。对多个中心机构注册研究的分析^[1, 4, 6-8]^表明，心肌炎的中位发病时间为开始治疗后17 d-65 d。Zhou等^[7]^回顾了既往发表的心脏不良事件病例，发现两种ICIs组合时心肌炎发生时间更早，53%的患者在开始治疗后4周内发生了心肌炎，而在单独应用ICI时，17%的患者在首剂ICI后发生了心肌炎，34%的患者在4个月后发生。这几项研究结果均表明心肌炎在ICIs治疗早期即可发生，两种ICIs联用时心肌炎发生时间更早。

危险因素尚不十分明确，除两种ICIs联合治疗增加心肌炎发生率外，研究^[2]^提示包括既往有自身免疫性疾病、基础心脏疾病、心力衰竭、急性冠脉综合征、糖尿病以及高龄等都可能会增加心肌炎的发生风险。

## 2 ICIs心肌炎病理特点及发病机制

### 2.1 ICIs相关心肌炎病理特点

ICIs相关心肌炎主要表现为多灶性或弥漫性或斑片状的淋巴细胞性心肌炎，其病理特点是心肌内CD3^+^ T淋巴细胞和CD68^+^巨噬细胞浸润，T淋巴细胞中含丰富的CD8^+^和CD4^+^ T细胞，传导系统也可受累，B细胞和抗体沉积明显缺乏，未见巨细胞浸润或肉芽肿，存在一定程度纤维化^[1, 2, 4, 7, 9-15]^。有研究^[14]^认为ICIs相关心肌炎以两种形式发生，一种为高等级（>50 CD3^+^细胞/hpf），其炎症细胞浸润增加，临床过程更急；另一种为低等级（≤50 CD3^+^细胞/hpf），其炎症细胞浸润程度较低，并且临床过程更加平缓。

### 2.2 ICIs相关心肌炎发病机制

ICIs相关心肌炎的具体机制尚不明确。近年来的研究^[16, 17]^表明，ICIs相关心肌炎可能存在多种机制，包括共有抗原、自身抗体、炎症因子的水平增加、T细胞自身反应、免疫耐受降低等。

#### 2.2.1 共有抗原

Johnson等^[1, 18]^对两例致命性心肌炎伴肌炎的黑色素瘤患者T细胞受体进行测序，发现心脏和肿瘤中浸润的淋巴细胞存在共享的高频T细胞受体序列，这表明接受ICIs治疗后激活的T淋巴细胞免疫应答不仅可以识别肿瘤细胞上的抗原，也可识别骨骼肌和心肌上的高同源抗原，从而诱发自身免疫性淋巴细胞性心肌炎。

#### 2.2.2 自身抗体

在临床前研究^[19]^中，当敲除MRL小鼠*PD-1*基因时，小鼠死于自身免疫性心肌炎，心脏T细胞被激活，同时产生抗心肌肌球蛋白的高滴度自身抗体。另外有研究显示敲除BALB/c小鼠中的*PD-1*基因可导致扩张性心肌病同时伴有严重收缩功能障碍和充血性心力衰竭，受累心脏显示高滴度IgG在心肌细胞表面弥漫性沉积，且存在针对心肌细胞表面表达的一种自身抗原的高滴度IgG自身抗体^[20]^。此外，在PD-1基因缺失的转基因小鼠模型中，由于高滴度的心肌肌钙蛋白I的自身抗体IgG增加了正常心肌细胞电压依赖性I型钙电流，导致进展为扩张型心肌病及过早死亡^[20]^。虽然临床前研究表明IgG在心肌细胞上沉积（识别心肌肌钙蛋白I的IgG），但人类ICIs相关的心肌炎似乎是由T细胞和巨噬细胞介导的，尚无证据表明B细胞或抗体沉积在心肌细胞上。

#### 2.2.3 炎性因子

除了T细胞和巨噬细胞的浸润外，还观察到多种趋化因子受体表达的变化，如肿瘤坏死因子α（tumor necrosis factor α, TNFα）、颗粒酶B和γ干扰素（interferon γ, IFN-γ）过度表达，表明细胞因子与免疫性心肌炎的发生也密切相关^[21, 22]^。在柯萨奇病毒B3诱导的心肌炎小鼠模型中，FrisanchoKiss等^[23]^研究发现CTLA-4以一种依赖于T细胞免疫球蛋白3的机制调控细胞群和心肌炎。Han等^[24]^应用CTLA-4激动剂治疗CVB3感染的小鼠，结果显示其IFN-γ水平降低，心肌炎发生率减少，生存率提高。另外一项研究^[25]^显示CTLA-4可调节心肌炎患者的CD8^+^ T细胞应答，单独CTLA-4或联合白介素-12诱导分化可协同降低CD8^+^ T细胞的心脏致病性。Tsuruda等^[26]^报道了3例ICIs治疗后发生心脏毒性的患者在不同阶段细胞因子的表达，1例经活检证实的心肌炎患者在发生Takotsubo心肌病时，白细胞介素（interleukin, IL）-8、单核细胞趋化激活因子和粒细胞巨噬细胞集落刺激因子（granulocyte-macrophage colony-stimulating factor, GM-CSF）水平升高，2例亚临床心肌炎患者在病程进展过程中IL-8激活，3例细胞因子释放综合征患者IL-6、IL-8、GM-CSF和IFN-γ大量激活。另外，最近有研究通过心肌活检转录组学发现炎症小体调节蛋白GBP5上调明显，表明NLRP3炎症小体可能参与了ICIs相关心肌炎的病理生理过程^[27]^。据报道，抗原呈递细胞（antigen-presenting cell, APC）的抗原呈递激活细胞毒性T淋巴细胞（cytotoxic T lymphocyte, CTL），然后抗原特异性CTL反馈激活APC中的NLRP3炎性体，形成一个正反馈回路，以放大CTL介导的抗肿瘤免疫^[28]^。

#### 2.2.4 自身免疫耐受下调

心肌细胞可能与肿瘤细胞一样利用PD-1/PD-L1和CTLA-4通路来防止T细胞在生理条件下过度活化。在许多临床前模型中，PD-1已被证明可以限制心脏中的T细胞反应，PD-1的敲除会引起CD8^+^ T细胞介导的自身免疫性扩张型心肌病和致命性心肌炎，表明PD-1对心肌炎和心脏损伤具有保护作用^[19, 20, 25]^。同样的，在PD-L1^-/-^MRL-Fas（lpr）小鼠的临床前模型中，PD-L1的敲除也会导致致死性自身免疫性心肌炎。另外，*PD-L2*基因缺失会加重实验性自身免疫性心肌炎小鼠的心肌炎症^[29]^。在成年小鼠敲除CTLA-4同样也会引起致命性的心肌炎、自发性淋巴增生、肺炎及胃炎等，并伴有器官特异性自身抗体，表明CTLA-4缺乏会影响中枢和外周耐受以及Treg细胞介导的抑制^[30]^。此外，在心脏损伤的患者中，PD-L1在受损心肌细胞上的表达上调。一致地，在T细胞介导的心肌炎的临床前研究中，PD-L1的表达也上调。PD -L1的上调可能保护心肌免受损伤，然而，这种上调可以被ICIs中和。综上所述，PD-1-PD-L1-PD-L2轴和CTLA4通路在限制T细胞介导的自身免疫性心肌炎中发挥重要作用。

#### 2.2.5 调节性T细胞稳态丧失

Läubli等^[31]^报道了1例Pembrolizumab介导的自身免疫性心肌炎发展为严重的心力衰竭的病例，心肌活检显示CD8^+^ T淋巴细胞浸润，而FOXP3^+^调节性T细胞减少。调节性T细胞（regulatory T cell, Treg）在维持免疫耐受和预防自身免疫方面发挥着至关重要的作用，irAEs的临床前模型显示Treg细胞数和irAEs之间呈负相关^[32]^。ICIs作用机制之一为Treg细胞的减少或清除^[33]^，ICIs治疗可能通过多种机制靶向Treg细胞，这种相互作用可导致Treg细胞的重新编程，将免疫平衡转向免疫紊乱导致irAEs的发生^[34]^。

### 2.3 临床前模型

然而，对irAEs机制的深入理解仍然存在着缺乏能概括临床过程的临床前动物模型这一主要障碍，为此，陆续有研究者开发了各种动物模型并探讨其中的机制。Ji等^[35]^开发了一种ICIs治疗后食蟹猴心肌炎模型，其特征是包括心肌炎在内的多种器官毒性，各器官轻中度的单核细胞浸润，心肌中的单核细胞浸润主要由T细胞组成，巨噬细胞较少，偶见B细胞，该模型中的心脏病变与临床报道的ICIs心肌炎相似，为疾病免疫机制的深入了解提供了平台。Liu等^[36]^从案例报道到小鼠模型验证了顺序或同时接受PD-1和PD-L1抑制剂治疗可能会导致严重的心脏毒性，在小鼠模型中PD-1阻断显著增加了心肌中的细胞毒性T细胞，仅在顺序阻滞的小鼠心肌中可见中性粒细胞浸润显著增加。Wei等^[37]^用Ctla4^+/-^ Pdcd1^-/-^小鼠模型的心脏病变概述了ICIs相关的自身免疫性心肌炎的临床过程，CTLA4-Ig干预可减轻心肌炎。Tsuruoka等^[38]^研究发现ICIs相关自身免疫性心肌炎的恶化取决于小鼠的给药时间，表明ICIs引起的自身免疫性心肌炎可能与接受ICIs治疗前自身免疫系统的活性有关。国内学者开展的一项研究中发现PD-1抗体影响巨噬细胞浸润和M1极化，可通过调节microRNA-34a/Kruppel样因子4信号转导巨噬细胞诱导心脏损伤^[39]^。

## 3 临床表现与诊断

ICIs相关的心脏毒性呈多样性，心肌炎是最常见的表现形式，还包括心包炎、心力衰竭、心律失常、Takotsubo综合征（心碎综合征）、传导功能障碍、心室功能损害等^[32, 40-42]^。临床表现各异，主要取决于病变的广泛程度和严重程度，少数可为无症状的心脏标志物升高，轻者表现为非特异性的轻微症状，重者表现为快速进展的心源性休克甚至致死性的暴发性心肌炎^[43]^。心肌炎是最常见的、致死率最高的心脏毒性，典型心肌炎综合征的临床表现包括乏力、气促、心悸、胸痛、急性或慢性心力衰竭、肺水肿、心源性休克、恶性心律失常等。另外，合并系统性自身免疫性疾病（如类风湿关节炎、红斑狼疮、银屑病关节炎、系统性硬化症等）的患者易发生亚临床心肌炎。诊断方法包括症状及体征、心电图、生物标志物（肌酸激酶同工酶、肌钙蛋白I、脑钠肽等）、心脏影像学检查（超声心动图及心脏磁共振），心肌活检是心肌炎确定诊断的金标准，免疫检查点抑制剂引起的心肌炎多见于黑色素瘤患者。

## 4 总结与展望

ICIs相关心肌炎是ICIs少见但严重的不良反应，具有发病早、症状无特异性、进展迅速、致死率高等特点，随着联合治疗的推行，ICIs的适应证不断扩大和治疗人群不断增多，其真实发生率可能会有所增加，临床医生需提高警惕。ICIs相关心肌炎的临床管理与治疗迫切需要规范化，合理管理并正视ICIs相关心肌炎的发生，尤其是早期识别和治疗，防止其进展为严重不良事件，有助于实现免疫治疗的最大临床效益。已有研究证实接受免疫治疗的患者发生irAEs可能预示着临床结局的改善，irAEs有望成为生存获益的预测指标，期待大型多中心临床研究进一步论证。ICIs相关心肌炎的发生机制目前尚不十分明确，应积极对其病理生理学、分子生物学机制等进行探索，以减少ICIs相关心肌炎的发生。除了临床前机制探索，如何预测和筛选高危人群、寻找可靠的生物标志物是未来研究的重点。

## References

[1] Johnson DB, Balko JM, Compton ML (2016). Fulminant myocarditis with combination immune checkpoint blockade. N Engl J Med.

[2] Mahmood SS, Fradley MG, Cohen JV (2018). Myocarditis in patientstreated with immune checkpoint inhibitors. J Am Coll Cardiol.

[3] Wang F, Sun X, Qin S (2020). A retrospective study of immune checkpoint inhibitor-associated myocarditis in a single center in China. Chin Clin Oncol.

[4] Salem JE, Manouchehri A, Moey M (2018). Cardiovascular toxicities associated with immune checkpoint inhibitors: an observational, retrospective, pharmacovigilance study. Lancet Oncol.

[5] Moslehi JJ, Salem JE, Sosman JA (2018). Increased reporting of fatal immune checkpoint inhibitor-associated myocarditis. Lancet.

[6] Neilan TG, Rothenberg ML, Amiri-Kordestani L (2018). Checkpoint inhibitor safety working group. myocarditis associated with immune checkpoint inhibitors: An expert consensus on data gaps and a call to action. Oncologist.

[7] Zhou YW, Zhu YJ, Wang MN (2019). Immune checkpoint inhibitor-associated cardiotoxicity: current understanding on its mechanism, diagnosis and management. Front Pharmacol.

[8] Hardy T, Yin M, Chavez JA (2020). Acute fatal myocarditis after a single dose of anti-PD-1 immunotherapy, autopsy findings: a case report. Cardiovasc Pathol.

[9] Tadokoro T, Keshino E, Makiyama A (2016). Acute lymphocytic myocarditis with anti-PD-1 antibody nivolumab. Circ Heart Fail.

[10] Sobol I, Chen CL, Mahmood SS (2020). Histopathologic characterization of myocarditis associated with immune checkpoint inhibitor therapy. Arch Pathol Lab Med.

[11] Ganatra S, Neilan TG (2018). Immune checkpoint inhibitor-associated myocarditis. Oncologist.

[12] Reuben A, Petaccia De Macedo M, Mcquade J (2017). Comparative immunologic characterization of autoimmune giant cell myocarditis with ipilimumab. Oncoimmunology.

[13] Zhang L, Awadalla M, Mahmood SS (2020). Cardiovascular magnetic resonance in immune checkpoint inhibitor-associated myocarditis. Eur Heart J.

[14] Champion SN, Stone JR (2020). Immune checkpoint inhibitor associated myocarditis occurs in both high-grade and low-grade forms. Mod Pathol.

[15] Ibraheim H, Perucha E, Powell N (2019). Pathology of immune-mediated tissue lesions following treatment with immune checkpoint inhibitors. Rheumatology (Oxford).

[16] Kennedy LB, Salama AKS (2020). A review of cancer immunotherapy toxicity. CA Cancer J Clin.

[17] Postow MA, Sidlow R, Hellmann MD (2018). Immune-related adverse events associated with immune checkpoint blockade. N Engl J Med.

[18] Berner F, Bomze D, Diem S (2019). Association of checkpoint inhibitor-induced toxic effects with shared cancer and tissue antigens in non-small cell lung cancer. JAMA Oncol.

[19] Wang J, Okazaki IM, Yoshida T (2010). PD-1 deficiency results in the development of fatal myocarditis in MRL mice. Int Immunol.

[20] Nishimura H, Okazaki T, Tanaka Y (2001). Autoimmune dilated cardiomyopathy in PD-1 receptor-deficient mice. Science.

[21] Sławiński G, Wrona A, Dąbrowska-Kugacka A (2020). Immune checkpoint inhibitors and cardiac toxicity in patients treated for non-small lung cancer: A review. Int J Mol Sci.

[22] Tocchetti CG, Galdiero MR, Varricchi G (2018). Cardiac toxicity in patients treated with immune checkpoint inhibitors: It is now time for cardio-immuno-oncology. J Am Coll Cardiol.

[23] Frisancho-Kiss S, Davis SE, Nyland JF (2007). Cutting edge: cross-regulation by TLR4 and T cell Ig mucin-3 determines sex differences in inflammatory heart disease. J Immunol.

[24] Han B, Jiang H, Liu Z (2012). CTLA4-Ig relieves inflammation in murine models of coxsackievirus B3-induced myocarditis. Can J Cardiol.

[25] Love VA, Grabie N, Duramad P (2007). CTLA-4 ablation and interleukin-12 driven differentiation synergistically augment cardiac pathogenicity of cytotoxic T lymphocytes. Circ Res.

[26] Tsuruda T, Yoshikawa N, Kai M (2021). The cytokine expression in patients with cardiac complication after immune checkpoint inhibitor therapy. Intern Med.

[27] Finke D, Heckmann MB, Salatzki J (2021). Comparative transcriptomics of immune checkpoint inhibitor myocarditis identifies guanylate binding protein 5 and 6 dysregulation. Cancers (Basel).

[28] Yao Y, Chen S, Cao M (2017). Antigen-specific CD8^+^ T cell feedback activates NLRP3 inflammasome in antigen-presenting cells through perforin. Nat Commun.

[29] Li S, Tajiri K, Murakoshi N (2021). Programmed death-ligand 2 deficiency exacerbates experimental autoimmune myocarditis in mice. Int J Mol Sci.

[30] Klocke K, Sakaguchi S, Holmdahl R (2016). Induction of autoimmune disease by deletion of CTLA-4 in mice in adulthood. Proc Natl Acad Sci U S A.

[31] Läubli H, Balmelli C, Bossard M (2015). Acute heart failure due to autoimmune myocarditis under pembrolizumab treatment for metastatic melanoma. J Immunother Cancer.

[32] Kumar P, Saini S, Prabhakar BS (2020). Cancer immunotherapy with check point inhibitor can cause autoimmune adverse events due to loss of Treg homeostasis. Semin Cancer Biol.

[33] Grabie N, Gotsman I, DaCosta R (2007). Endothelial programmed death-1 ligand 1 (PD-L1) regulates CD8^+^ T-cell mediated injury in the heart. Circulation.

[34] Alissafi T, Hatzioannou A, Legaki AI (2019). Balancing cancer immunotherapy and immune-related adverse events: The emerging role of regulatory T cells. J Autoimmun.

[35] Ji C, Roy MD, Golas J (2019). Myocarditis in cynomolgus monkeys following treatment with immune checkpoint inhibitors. Clin Cancer Res.

[36] Liu SY, Huang WC, Yeh HI (2019). Sequential blockade of PD-1 and PD-L1 causes fulminant cardiotoxicity-from case report to mouse model validation. Cancers (Basel).

[37] Wei SC, Meijers WC, Axelrod ML (2021). A Genetic mouse model recapitulates immune checkpoint inhibitor-associated myocarditis and supports a mechanism-based therapeutic intervention. Cancer Discov.

[38] Tsuruoka K, Wakabayashi S, Morihara H (2020). Exacerbation of autoimmune myocarditis by an immune checkpoint inhibitor is dependent on its time of administration in mice. Int J Cardiol.

[39] Xia W, Zou C, Chen H (2020). Immune checkpoint inhibitor induces cardiac injury through polarizing macrophages via modulating microRNA-34a/Kruppel-like factor 4 signaling. Cell Death Dis.

[40] Hu JR, Florido R, Lipson EJ (2019). Cardiovascular toxicities associated with immune checkpoint inhibitors. Cardiovasc Res.

[41] Ball S, Ghosh RK, Wongsaengsak S (2019). Cardiovascular toxicities of immune checkpoint inhibitors: JACC review topic of the week. J Am Coll Cardiol.

[42] Bonaca MP, Olenchock BA, Salem JE (2019). Myocarditis in the setting of cancer therapeutics: proposed case definitions for emerging clinical syndromes in cardio-oncology. Circulation.

[43] Escudier M, Cautela J, Malissen N (2017). Clinical Features, Management, and Outcomes of Immune Checkpoint Inhibitor-Related Cardiotoxicity. Circulation.

